# Editorial: Post-translational mechanisms involved in regulating peroxisome biogenesis, functions and organelle-crosstalk

**DOI:** 10.3389/fcell.2023.1136992

**Published:** 2023-01-12

**Authors:** Amr Kataya, Eric Fedosejevs, Yajin Ye

**Affiliations:** ^1^ Department of Biological Sciences, University of Calgary, Calgary, AB, Canada; ^2^ Harrow Research and Development Centre, Agriculture and Agri-Food Canada, Harrow, ON, Canada; ^3^ College of Forestry, Nanjing Forestry University, Nanjing, China

**Keywords:** peroxisomes, signaling, post-translational modification (PTM), phosphorylation, sulfenylation, Zellweger syndrome, pexophagy, hydrogen peroxide signaling

This Research Topic, on post-translational mechanisms involved in regulating peroxisome biogenesis, functions, and organelle-crosstalk, covers control mechanisms of peroxisome dynamics and biogenesis in plants, yeast, and human. Peroxisome biogenesis, roles, and crosstalk are expected to be fine-tuned under varying cellular conditions for example when exposed to environmental stresses, and between various developmental stages and tissues. This requires highly dynamic and versatile processes such as post-translational modifications (PTM) and complex signaling. Identifying these factors and their mechanistic regulation of peroxisome biogenesis and roles will offer new insights into basic sciences.

The Research Topic presents six articles including two original research and reviews on peroxisome-related research. We will represent in this editorial the aspects of these articles. Phosphorylation, ubiquitination, acetylation, and asparagine (N)-linked glycosylation PTMs were found associated and/or predicted with peroxisomal proteins (Sandalio et al., 2019; Infant et al., 2021). In the past decade, advanced proteomics helped to identify a huge number of PTMs such as phosphorylated peroxisomal proteins in several organisms ((Oeljeklaus et al., 2016; Kataya et al., 2019). A limited number of studies have identified functional regulation of PTMs for example, yeast glycerol-3-phosphate dehydrogenase 1 was found to change its localization to peroxisomes through phosphorylation of two serine residues downstream of its peroxisome targeting signal type 2 (PTS2) Ohsawa et al. In this Research Topic have reviewed the recent findings highlighting the regulation of peroxisome homeostasis in the methylotrophic yeast *Komagataella phaffii* Ohsawa et al. Methanol allows the yeast to grow with peroxisome proliferation that subsequently degrades by pexophagy upon methanol depletion, a mechanism that is repressed by the addition of ethanol. The authors discussed PTM-based signaling, where phosphorylation has been shown to inactivate Mxr1 transcription factor during ethanol repression and to trigger peroxisome-membrane-associated Atg30 to activate pexophagy under methanol depletion. The authors also have discussed how pexophagy is repressed during methanol signaling and the physiological significance of these types of machinery.

In plants, only the photorespiratory enzyme, glycolate oxidase activity has been shown to be regulated by phosphorylation *in vitro* and its phospho-status differentiated in response to light and CO_2_ (Dellero et al., 2013; Jossier et al., 2019). So far, a limited number of protein kinases were identified in peroxisomes of humans, plants, and yeast, and three protein phosphatases were reported only in plant peroxisomes with little knowledge of their functions (reviewed in (Kataya et al., 2019)). In this Research Topic: original research by Kataya et al. has investigated bioinformatically peroxisome kinome in the model organism “*Arabidopsis thaliana*” for the presence of putative functional PTS type 1 (PTS1), and identified a considerable number of protein kinases that putatively harbors functional PTS1. The fact that the Arabidopsis genome encodes up to 1,000 protein kinases and the identified protein kinases conservation of PTS1-like tripeptides aligned with experimental verification of 12 functional PTS1s. This was accomplished by the authors by fusing protein kinases terminal domains with EYFP and investigating their targeting into peroxisome *in vivo*. This study also investigated if calcium-dependent protein kinase 1 (CDPK1) and glyoxysomal protein kinase 1 (GPK1), which were previously reported as protein kinases (reviewed in (Kataya et al., 2019)), can target peroxisomes in a PTS1-dependent fashion. In addition, the study successfully reported the ability of seven new full-length protein kinases to target peroxisomes *in vivo* and investigated their impact on peroxisomal fatty acid β-oxidation in the protein kinases isolated T-DNA mutants. Overall, this research opens more avenues to study the effect of protein phosphorylation on peroxisome functions and should help identify functional substrates involved in peroxisome signaling.

As described above, the advancements in bioinformatics and predictive models help in identifying novel and rare PTSs or in other terms canonical and non-canonical PTSs. Kataya et al., used PTS1-developed models by (Lingner et al., 2011; Wang et al., 2017) to identify new PTS1s in Arabidopsis protein kinases. However, as Tarafdar and Chowdhary emphasized in their review of this Research Topic, investigating the peroxisome proteome of economically important crops was not fully investigated. In their article, a reference peroxisomal matrix proteome map for *Arabidopsis thaliana* was generated including reported proteomic, bioinformatic predicted, and experimentally verified peroxisomal proteins. Employing this reference map, Tarafdar and Chowdhary identified putative peroxisome proteome in *S. lycopersicum*, which showed greater diversity in the composition of the signal tripeptide. These comparative studies are timely needed and should facilitate future studies investigating peroxisome signaling in such an important crop.

The generation and signaling of peroxisomal reactive oxygen species are expected to be fine-tuned. The thiol side chain (RSH) of Cys is a major target for reactive oxygen species that can be sulfenylated (Cys-SOH). Scarce information is present about the targets of peroxisomal hydrogen peroxide and the regulatory role of peroxisomes involving reactive oxygen species in peroxisomal signaling. Lismont et al. in this Research Topic, have shown the sulfenylation profiles of key redox signaling proteins are indeed affected by peroxisome-derived hydrogen peroxide. The authors employed a cell system, Flp-In T-REx 293, where they can modulate peroxisomal hydrogen peroxide in combination with a yeast AP-1-like-based sulfenome mining strategy and identified around 400 thiol targets. These targets, as reported, are derived in response to peroxisome-derived hydrogen peroxide and reside not only in peroxisomes but also in cytosol and mitochondria. The sulfenylation profiles and kinetics in response to peroxisome-derived hydrogen peroxide have shown considerable differentiation of peroxiredoxins and tubulins. Moreover, the authors have shown putative evidence of the involvement of the redox-relay mechanism to oxidize peroxisomal hydrogen peroxide targets such as transcription factors. These results take one step forward in understanding how peroxisomes interact with cell constituents and integrate into the cellular hydrogen signaling network.

Peroxisome mutants in humans cause peroxisomal biogenesis disorders such as Zellweger syndrome which is a metabolic disorder with severe pathology in multiple organs with little known about pathogenesis. Jiang and Okazaki in this Research Topic have reviewed recent findings and discussed how peroxisomes are involved in regulating intrinsic apoptotic pathways and upstream fission-fusion processes that could cause multiple organ dysfunctions of Zellweger syndrome. The authors also provided mammalian cells-based findings implicating the regulatory roles of peroxisomes in mitochondrial fission-fusion dynamics and intrinsic apoptotic pathways through redox control.

Peroxisomes are known to be highly dynamic as they proliferate by *de novo* biosynthesis and division, can increase in number due to environmental stresses, and extend to form peroxules to interact with other organelles. This versatility in peroxisome morphological appearances requires complex signaling that putatively includes PTMs. For example, peroxisomes proliferation has been shown to be influenced by the protein kinase MPK17 (Frick and Shrader, 2018) due to salt stress. Salt stress also has been shown to affect the expression of peroxisomal genes in a process that requires ethylene, jasmonate, and abscisic acid signaling pathways (Charlton et al., 2005). Also, in an intriguing study, peroxisomes were found to extend peroxules in response to stress *via* reactive oxygen species involving the induction of the peroxin 11a, which leads to higher proliferation and execute its proliferation (Rodríguez-Serrano et al., 2016). The need to systematically study these peroxisome features is crucial, and the Goto-Yamada et al. review article on this Research Topic has thoroughly investigated molecular dynamics or peroxisomes in plants through image-based analysis. The authors have addressed and compared molecular mechanisms that putatively regulate peroxisomes dynamics and morphological appearances utilizing fluorescence imaging, especially in *Arabidopsis thaliana* aberrant peroxisome morphology (*apem*) and peroxisome unusual positioning (*peup*) mutants and the liverwort *Marchantia polymorpha* that is emerging as useful plant model. They have analyzed peroxisome biogenesis, proliferation and quality control, and physical organelle-organelle interactions and conclude that *Marchantia polymorpha* is a useful organism to identify genes bioinformatically, mutate, and investigate dynamics and diversity of peroxisomes in land plants. The authors also highlighted the demanding need to systematically screen and phenotypically investigate large number of mutants. Therefore, they have highlighted the study by Li et al., which categorized mutant phenotypes and identified abnormal morphology using a newly established Deep Learning of the Morphology of Organelles (DeepLearnMOR) tool (Li et al., 2021). Taken together, these advances in studying molecular mechanisms regulating peroxisome dynamics will facilitate future studies investigating PTM involvement.

The PTM investigation in peroxisomes remains largely unknown. This Research Topic, for the first time, specifically focused on the regulation of peroxisomes by PTMs. This Research Topic covered studies in plants, yeast, and human peroxisomes, discussed and supplied evidence of PTM-dependent regulation of peroxisome proteome, the presence of peroxisome-PTM modifiers, and the putative regulatory effect of peroxisomes on mitochondrial biogenesis and pathogenesis. Moreover, utilizing bioinformatic and prediction algorithms and image-based analyses were thoroughly discussed which will help future analyses of this research field.
